# Metacognitive training facilitates optimal cognitive offloading

**DOI:** 10.1186/s41235-026-00714-0

**Published:** 2026-03-12

**Authors:** Ceri Ngai, Sam J. Gilbert

**Affiliations:** Institute of Cognitive Neuroscience, University College London, 17 Queen Square, London, WC1N 3AZ UK

**Keywords:** Cognitive offloading, Intention offloading, Decision-making, Metacognition, Memory

## Abstract

Cognitive offloading refers to the use of physical actions and the external environment to reduce cognitive demand. Offloading strategies such as creating external reminders instead of relying on internal memory are highly effective and play a key role in supporting real-world cognition. Previous work has shown that people have systematic biases in their offloading strategies, which are related to biased metacognitive evaluations of cognitive ability. While metacognitive interventions could potentially mitigate these biases, research investigating their effects has produced mixed results. Here, we examined the influence of a brief metacognitive intervention comprising just five trials during an initial practice session. After the intervention, participants performed a memory task where they decided between using internal memory (for maximum reward) or external reminders (for reduced reward), allowing us to determine the optimality of offloading strategies. Experiment 1 (*N* = 164) showed that making metacognitive predictions and subsequently receiving feedback led to improved metacognitive calibration and more optimal reminder-setting strategies. Experiment 2 (*N* = 416) replicated this pattern and found that making predictions alone was ineffective. These findings suggest that a metacognitive intervention combining prediction with feedback could potentially optimise cognitive offloading in everyday life.

## Significance statement

People often use external tools and devices to extend their cognitive abilities. For example, if you have to remember to do something, you might set a reminder or alert in your smartphone rather than just remembering it yourself. This is known as cognitive offloading. Previous research has shown that people do not always use external devices optimally: some people set more reminders than they need, others do not set enough. This means that it is important to find ways of helping people use tools more effectively for tasks like remembering to take medication or attending appointments. A key factor that determines cognitive offloading is *confidence*: people tend to set reminders when they *think* that they may forget something, but beliefs about memory ability do not necessarily match reality. Here, we tested 1) whether we can improve people’s insight into their true memory capacity, and 2) whether this alters their use of reminders. Across two experiments we gave people a brief intervention where they had to predict how well they would remember some information, and then, we told them how well they had actually remembered. We found that doing this just five times made people more accurate in their predictions for how well they would remember in the future. We also found that this made them more optimal in their future choices about whether to set reminders. The greatest improvements were seen when people not only made predictions but also received feedback. This suggests that an intervention combining performance prediction with feedback could help people to judge their memory ability more accurately and improve everyday use of memory aids.

## Introduction

From penning down an appointment in one’s diary, setting up an automatic reminder so that you receive a notification the day before, to adding new items to your to-do list before you forget, examples of ‘intention offloading’ are abundant in many aspects of everyday life. Intention offloading refers to the creation of external reminders or cues in the environment to help remember a future intention (Gilbert et al., 2023). This is an example of the more general phenomenon of ‘cognitive offloading’, the use of one’s body, physical action, and the external environment as a means of reducing cognitive demands (Risko & Gilbert, 2016).

Intention offloading offers considerable benefits for everyday cognitive functioning. Such benefits are evident not only in experimental tasks, where offloading strategies can reduce memory errors by an order of magnitude or more (Gilbert et al., 2020), but also in naturalistic and real-world studies of everyday memory, which have shown strong improvements in memory performance when external aids are used (Jones et al., 2021; Scott & Gilbert, 2024; Scullin et al., 2021).

However, offloading also comes with potential costs. On a practical level, creating external reminders requires time and effort. Although the individual cost of setting a reminder is often small, these costs could accumulate to an unacceptable level if people set reminders for every routine activity that would have been remembered unaided. Therefore, some selection process is required, rather than indiscriminately offloading all cognitive demands to the external environment (Gilbert, 2024). In the longer term, excessive use of external tools may also contribute to declines in unaided cognitive abilities. For example, reliance on cognitive offloading may reduce internal memory for to-be-remembered information (Sparrow et al., 2011), and excessive use of tools such as GPS devices could potentially lead to a decline in navigation abilities (Dahmani & Bohbot, 2020; Hejtmánek et al., 2018).

Seeing as cognitive offloading carries both potential benefits and risks, research has aimed to investigate 1) whether people are biased, relative to an optimal strategy, in their use of cognitive offloading, and 2) whether interventions can be found to mitigate such biases. Improving offloading strategies in this way could promote more effective and adaptive use of cognitive tools in everyday life.

One way in which the optimality of cognitive offloading can be measured is with the ‘optimal offloading’ paradigm introduced by Gilbert et al. (2020). In this paradigm, participants perform a demanding memory task in which they decide between using internal memory (in which case they earn maximum reward for each remembered item) or external reminders (in which case they earn a reduced reward that varies from trial to trial). As a result, participants need to balance the improved accuracy brought by external reminders against the reduced reward. By comparing participants’ strategy choices with the optimal strategy, which can be objectively determined from their performance measures, bias in cognitive offloading can be objectively quantified. Studies using this paradigm consistently find that participants are biased towards excessive use of reminders, meaning that they tend to choose the external reminder option even when they would have earned more points (and, in some studies, greater financial reward) with internal memory (Ball et al., 2022; Fröscher et al., 2022; Gilbert et al., 2020; Kirk et al., 2021; Sachdeva & Gilbert, 2020, 2024). Moreover, individual differences in reminder bias remain stable over time (Gilbert et al., 2020). This raises an important question: how might such biases be mitigated?

### Metacognition as a target for interventions

Understanding the causes of offloading is crucial for designing interventions which target such biases. Previous work has repeatedly shown that a key factor in determining people’s decisions to offload is *confidence* in one’s memory abilities: the less confident they are, the more likely they are to set external reminders (Boldt & Gilbert, 2019; Gilbert, 2015; Gilbert et al., 2020; Sachdeva & Gilbert, 2024; reviewed by Gilbert et al., 2023). Importantly, this link between confidence and offloading is evident even when objective memory ability is controlled for, or when confidence is entirely unrelated to actual memory performance (Gilbert, 2015). This pattern holds both when participants are explicitly instructed that they can set reminders if they want to (e.g. Gilbert et al., 2020), and when they have to spontaneously generate the offloading strategy themselves (Boldt & Gilbert, 2019). Beyond laboratory tasks, confidence in memory ability has also been found to predict offloading in real-life settings—for instance, when participants’ self-generated intentions are embedded within their daily activities (Scott & Gilbert, 2024). Thus, offloading is guided not only by one’s objective memory ability, but also by metacognitive beliefs about one’s ability to remember.

An obvious corollary from these findings is that interventions which alter metacognitive beliefs may also influence offloading strategies. Initial evidence in support of this hypothesis comes from Gilbert et al., (2020, Experiment 3), who showed that manipulating people’s confidence in their own memory led to parallel shifts in offloading strategies. Specifically, the study used a 2 × 2 between-subject design crossing feedback valence (positive, negative) and practice difficulty (easy, difficult). Participants who received positively framed feedback and/or easier practice trials reported higher confidence in their own memory. Crucially, increased confidence led to reduced reminder setting (and vice versa), even though the interventions had no effect on objective memory performance. Mediation analyses further confirmed that shifts in offloading strategies were significantly mediated via shifts in confidence. Together, these findings demonstrate that it is possible to up- or down-regulate confidence, and that this in turn down- or up-regulates reminder setting.

However, other studies investigating the influence of metacognitive interventions have produced inconsistent results. For example, a recent study by Grinschgl et al. (2020) examined whether manipulating participants’ beliefs about their own working memory ability influences their propensity to offload. Participants were presented with fake performance feedback indicating how well they performed on three working memory tasks relative to their peers (either above- or below-average; the control group received no feedback). Although this intervention successfully altered participants’ confidence in their working memory abilities, it had no effect on their offloading behaviour during a Pattern Copy Task (Ballard et al., 1997), suggesting that changes in metacognitive beliefs do not always translate to changes in offloading strategies. Furthermore, in practical terms, the most useful form of metacognitive intervention may not be one that always increases or decreases confidence, but rather one that improves the *calibration* of metacognitive judgements. Achieving this may require increasing confidence for some individuals and decreasing it in others.

Studies on metacognitive training show that interventions aimed at improving the calibration of metacognitive judgements can indeed be effective (Adams & Adams, 1958; Baird et al., 2014; Carpenter et al., 2019; Sella et al., 2023; Sharp et al., 1988). In the context of perceptual decision-making, Carpenter et al. (2019) found that providing feedback based on participants’ metacognitive judgements—rather than on their actual task performance—improved their metacognitive accuracy. Interestingly, this improvement in accuracy generalised both to untrained stimuli and to an untrained recognition memory task (though see Rouy et al., 2022, for a critique of these findings).

To test whether a similar intervention could increase the optimality of offloading judgements, Engeler and Gilbert (2020) administered a brief metacognitive intervention before the optimal offloading task. During a nine-trial training session, participants in the feedback group made metacognitive predictions on how well they thought they could perform the task on each trial, and then received feedback indicating whether their prediction was underconfident, overconfident, or accurately calibrated. Results showed that the intervention was effective in improving metacognitive calibration, with the feedback group demonstrating significantly more accurate metacognitive judgements than the control group. However, when participants subsequently completed the optimal offloading task, there was no significant difference in offloading strategies between groups; both groups exhibited a bias towards excessive reminder use relative to the optimal strategy. Therefore, although the metacognitive intervention improved self-estimates, it did not produce downstream effects on offloading strategies.

In sum, while existing evidence shows that interventions targeting metacognition can influence confidence judgements and enhance metacognitive calibration, evidence remains inconclusive as to whether such improvements always translate to changes in offloading behaviour. Given the potential benefits and costs associated with offloading, identifying interventions that promote more optimal reminder use could have significant practical implications.

## Experiment 1

The purpose of this study was to repeat the metacognitive intervention procedure of Engeler and Gilbert (2020) with two main adjustments that could potentially uncover a link between metacognitive training and more optimal cognitive offloading. The first of these adjustments was that we provided financial reward to incentivise optimal cognitive offloading, unlike Engeler and Gilbert (2020). Previous research has suggested that participants may be biased towards excessive offloading not only due to inaccurate metacognitive beliefs, but also due to a preference to avoid the cognitive effort of remembering. This preference can be reduced by offering participants a financial reward determined by the optimality of their offloading strategies (Sachdeva & Gilbert, 2020). Therefore, providing a financial incentive for optimal cognitive offloading should reduce the influence of non-metacognitive factors on offloading strategies and hence make it more likely that the influence of metacognitive interventions would be observed. Whereas some previous studies have rewarded participants by paying them directly in proportion to the number of points scored in the task (e.g. Gilbert et al., 2020; Sachdeva & Gilbert, 2020), here we did this by simply paying a small bonus to participants with scores in the top 50%. Note that this incentive does not necessarily reward greater offloading or greater use of internal memory; instead, it rewards a decision-making strategy that most closely matches the optimal policy, based on each participant’s accuracy when they perform with one or the other strategy.

The second adjustment to the procedure of Engeler and Gilbert (2020) was that we used an updated optimal offloading paradigm that could provide a more sensitive measurement of bias in offloading strategies, making it more likely to observe any influence of metacognitive training if such an influence exists. We investigated two key hypotheses. The first was that our metacognitive intervention would reduce metacognitive bias, defined as the discrepancy between participants’ beliefs about their task performance and their true level of performance. The second was that our metacognitive intervention would reduce reminder bias, defined as the discrepancy between each participant’s reminder-setting strategy and their individually calculated optimal strategy.

### Methods

#### Design

We used a between-group design, comparing a group of participants receiving a metacognitive intervention during the practice phase (feedback group) with a group of participants that did not (no-feedback group). The hypotheses, experimental procedures, and analyses plans for this study were pre-registered prior to data collection (https://osf.io/8yrew/).

#### Participants

Participants were recruited via the Prolific website (https://www.prolific.co) and took part by accessing a weblink on their own computer. They received a base payment of £2.75 for taking part, and those who scored in the top 50% on the experimental task received an additional £1 bonus. All participants gave informed consent, and the research was approved by the UCL Research Ethics committee (1584/002).

A total of 164 participants were included in the study (age range: 18–79, mean age = 35.3, SD = 14.1; 56 women, 103 men, 5 other genders). The sample size was derived from a power calculation based on Engeler and Gilbert’s (2020) study, which found a significant reduction in internal metacognitive bias in the feedback group (*d* = 0.78). We decided to power this study so that it would have adequate power to detect a reduction in reminder bias with half of this magnitude. Specifically, we conducted a power calculation based on an independent sample t-test, one-tailed (based on the prediction that reminder bias would be reduced in the feedback group), with 80% power to detect an effect size of *d* = 0.39. This yielded a sample size of 164 (82 in each group, G*Power 3.1.9.6). We tested a total of 189 participants to reach the planned sample size after applying our pre-registered exclusion criteria. These criteria were: (a) forced internal accuracy below 10% (n = 0), (b) forced external accuracy below 70% (n = 11), (c) negative correlation between target value and likelihood of choosing to use reminders, suggesting random or counter-rational strategy choice (n = 6), (d) higher accuracy on forced internal than forced external trials (n = 1), (e) reminder bias score more than 2.5 Median Absolute Deviation units from other participants within their group (n = 5), (f) metacognitive bias score more than 2.5 Median Absolute Deviation units from other participants within their group (n = 2).

#### Updated optimal offloading task

The main experimental task was based on the optimal offloading paradigm first introduced by Gilbert et al. (2020), with some modifications intended to improve its sensitivity while reducing the testing duration.

**Fig. 1:**
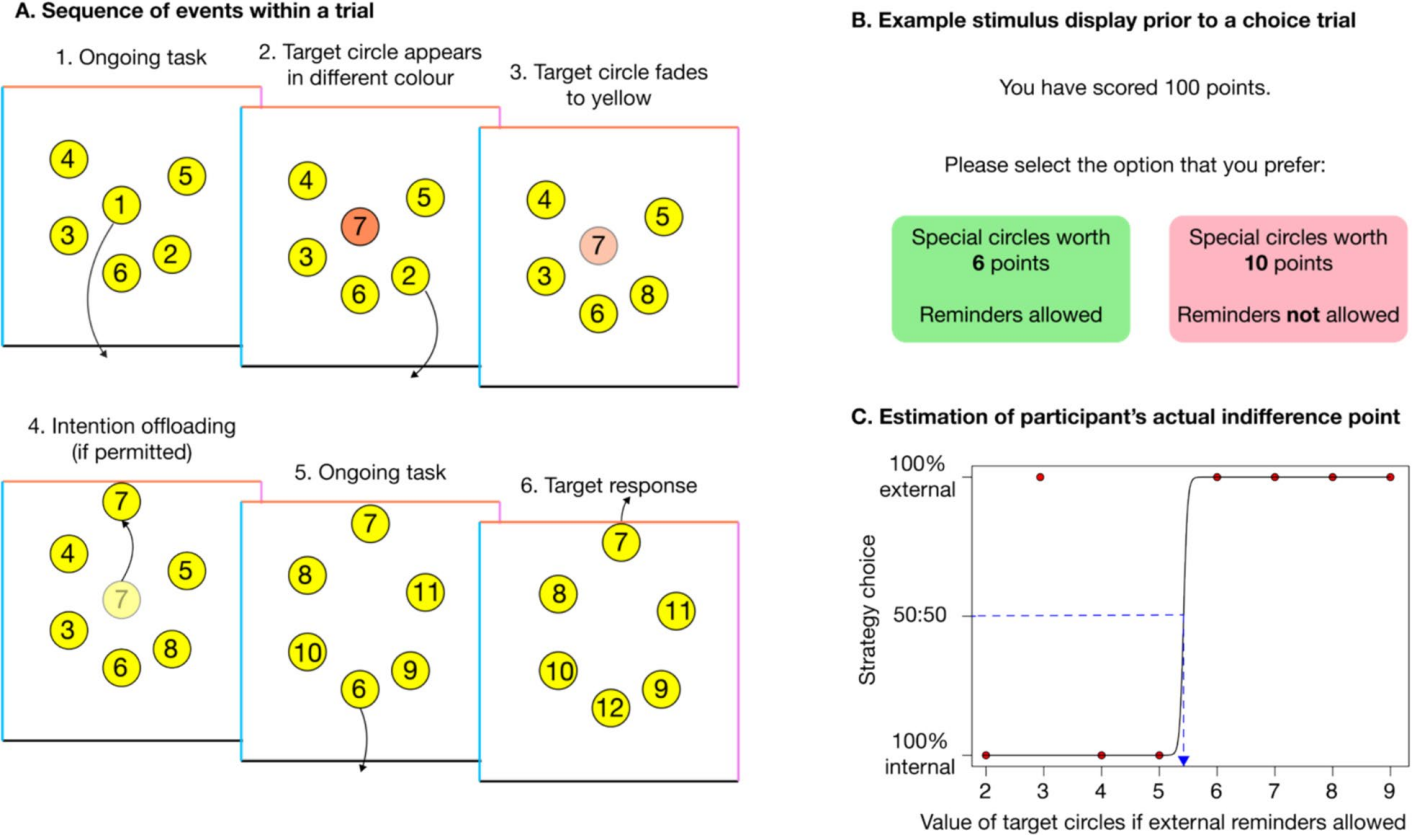
Schematic illustration of the experimental task, adapted from Gilbert et al. (2020)

On each trial, six numbered circles were shown inside a box which had its bottom, left, top, and right edges coloured black, blue, orange, and pink, respectively (Fig. 1). The participant used their mouse or touchscreen tablet to drag the circles in numerical order to the bottom of the box, which removed them from the screen. Each time a circle was removed from the screen, a new one appeared in its place to continue the sequence (e.g. after dragging the ‘1’ circle to the bottom, a new ‘7’ circle appeared in its place; then, after dragging the ‘2’ to the bottom it was replaced with an ‘8’, and so on). The circles were yellow, but sometimes when a new circle appeared on the screen, it was initially coloured blue, orange, or pink, before fading to yellow after 2 s. This was an instruction that when it was reached in the sequence, it should be dragged to the side of the box with the corresponding colour, instead of the bottom. For example, a participant might drag the ‘1’ to the bottom of the box, after which it could be replaced with a blue ‘7’ that faded to yellow after 2 s. Meanwhile, they would sequentially drag the circles labelled 2–6 to the bottom of the box, before dragging the 7 to the left as instructed by its initial (but no longer visible) colour.

Participants could perform this task either by relying on internal memory (e.g. just remember that the 7 needs to be dragged to the left) or external reminders, in which case they would immediately drag the target circles next to the instructed side of the box as soon as they first appeared. Their location would then act as a reminder for where they should eventually be dragged. In this case, there was no need to maintain an internal representation of the intended action because the location of the circle acted as an external reminder, like leaving an object next to the front door so that you remember to take it with you when leaving home the next day.

There were three types of trials in this task: on ‘choice trials’, participants were given the option of A) remembering the target circles with internal memory, in which case each correct response was worth 10 points, or B) using external reminders, in which case the target circles were worth a smaller number of points that varied from trial to trial. For example, participants might choose between earning 10 points for each correct target response with internal memory, or 6 points for each correct target response with external reminders. On ‘forced internal’ trials, participants were forced to use their own memory and on ‘forced external’ trials participants were forced to use external reminders. They were forced to use the selected strategy because, in the internal condition, all circles were fixed in place apart from the upcoming one in the sequence (e.g. if circles 2–7 were on the screen, the participant could only move the ‘2’ so they would not be able to set a reminder for the ‘7’); in the external condition participants were required to adjust the position of any target circles before they were allowed to continue the task.

Based on accuracy levels in the forced internal and external trials, it is possible to calculate the value attached to targets in the choice trials where an unbiased participant should be indifferent between the two strategies. For example, if an unbiased participant averaged 60% accuracy using their own memory and 100% accuracy using reminders, then they should be indifferent between earning 10 points per target using their own memory or 6 points per target using reminders. Based on actual decisions during the choice trials, the actual point of indifference between the two strategies can be calculated. The reminder bias can then be calculated as the difference between the optimal indifference point (based on accuracy in the forced internal and external conditions) and the actual indifference point (based on strategy choices collected before choice trials).

The modifications from the paradigm used in previous studies (Engeler & Gilbert, 2020; Gilbert et al., 2020) were as follows. First, whereas each trial of the original task had 25 circles with 10 targets embedded, in this study there were a total of 15 circles to remove from the box, with 5 target circles embedded within this sequence. This helped reduce the duration of the task. The target circles were distributed as evenly as possible between the circles numbered 7–15 (no targets could be presented for circles 1–6 because these are already on screen at the beginning of the trial), with one randomly selected side of the box receiving 1 target and the other two sides receiving 2 targets each.

Second, the original version of the task included 9 choice trials (with target values from 1–9 sampled once), and 8 forced trials (where no strategy choices were made). The choice trials were used to calculate the actual indifference point between the two strategies (based on strategy selections, discarding subsequent task performance). The forced trials were used to calculate the optimal indifference point (based on task performance; there were no strategy selections on these trials). This is inefficient because each type of trial only contributes one type of data: either strategy selection or task performance.

In the revised paradigm used here, there were 16 trials in total and participants were not informed of any distinction between choice and forced trials. They made a strategy choice on every trial, with target values from 2–9 each presented twice. Within the 16 trials, four trials were designated as forced internal and four as forced external. On these trials, if the participant’s choice differed from the designated condition (e.g. choosing to use reminders on a forced internal trial), they were informed that their choice had been ‘overwritten’ by the computer and that they would have to use the strategy selected by the computer instead. Because only half of the trials were forced, and choices on the forced trials were only overwritten if there was a mismatch between the chosen and the designated condition, participants performed their chosen strategy on a majority of trials. Trials were presented in a random order, with the constraint that each target value from 2–9 was presented once (in random order) and then once again (re-randomised). Furthermore, target values of 2,4,6,8 were assigned to forced internal and values 3,5,7,9 to forced external, or vice versa (randomised between participants).

The final modification was that the choice trials, which contributed strategy choice data but were not used to assess performance of the actual task, were terminated early. Participants simply dragged the initial 6 circles to the bottom of the box and then the trial ended. This helped keep the task duration to a minimum. Seeing as these trials were intermixed with forced internal and forced external trials, participants could not predict which trials would end early and which would not.

#### Procedure

Participants were introduced to the task and instructed to practice dragging 10 circles (with no targets) in sequential order to the bottom of the box. Target circles were then introduced, and participants completed two practice trials at increasing levels of difficulty: first, a trial with 10 circles and 3 targets; second, a trial with 15 circles and 5 targets.

#### Feedback manipulation

After this, participants performed further practice trials and the metacognitive intervention was delivered for the feedback group but not the no-feedback group. Participants performed 5 trials of the forced internal condition, with 15 circles and 5 targets. Before each trial, participants in the feedback group were given the following instructions: 'Before the next practice trial we would like you to tell us how **confident** you are that you can accurately perform the task. Please use the slider below to indicate what percentage of the special circles you will correctly drag to the instructed side. 100% would mean that you always get every single one correct. 0% would mean you can never get any of them correct'. They then completed one trial, followed by metacognitive feedback in the following form: 'You predicted that you will get X% correct. You actually got Y% correct. You underestimated / overestimated / accurately estimated your memory ability'. This prediction, performance, and feedback sequence was repeated 5 times. Participants in the no-feedback group also completed 5 forced internal practice trials, but they did not make metacognitive predictions beforehand or receive feedback.

#### Pre-task metacognitive judgement

Following this 5-trial practice session, participants in both groups provided a prospective metacognitive confidence judgement. They were asked: 'Before continuing, we would like you to tell us how **confident** you are that you can accurately perform the task for the rest of the experiment. Please use the scale below to indicate what percentage of the special circles you can correctly drag to the instructed side of the square, on average. 100% would mean that you always get every single one correct. 0% would mean that you can never get any of them correct'.

#### Final practice trials and experimental block

After this judgement, participants received instructions on how to set external reminders and performed one trial in the forced external condition. They then practised making decisions between the two strategies, each with an associated point value for targets, and were instructed about the occasional ‘overwriting’ of their chosen strategy by the computer. Finally, they received instructions explaining that some trials would end early, before any targets were presented. After this, they performed the full experimental block of 16 trials.

#### Final metacognitive judgement

Following the experimental trials, participants provided a final metacognitive judgement with the instructions: 'Last of all, we would like to ask you one last time about your **confidence**. Suppose you had to do the task again using your **own memory**, without being able to use reminders. What percentage of the special circles do you think you would be able to correctly drag to the instructed side of the square, on average? 100% would mean that you always get every single one correct. 0% would mean that you could never get any of them correct. Please remember that you should just answer about your ability to do the task with your **own memory**'. After this, they were debriefed, thanked, and paid.

#### Data analysis

Analyses were performed in R (version 4.2.1) and RStudio (version 2022.07.24576). All statistical analyses were conducted in accordance with our preregistration, which can be found along with data and code at https://osf.io/g45aq, except where noted. The key dependent variables were the same as those in Engeler and Gilbert (2020), with the exception that we did not collect metacognitive judgements about the external condition in this study. Measures were calculated as follows:

- Forced internal accuracy (ACC_FI_): Mean accuracy of dragging target circles to the instructed location on forced internal trials.
- Forced external accuracy (ACC_FE_): Mean accuracy of dragging target circles to the instructed location on forced external trials.
- Optimal indifference point (OIP): Value (2–9) at which an unbiased individual would be indifferent between earning this number of points with external reminders or 10 points with internal memory. As in Gilbert et al. (2020) this is computed as:
  $${\mathrm{OIP}} = \frac{{10*{\mathrm{ACC}}_{{{\mathrm{FI}}}} }}{{{\mathrm{ACC}}_{{{\mathrm{FE}}}} }}$$
- Actual indifference point (AIP): Actual value at which participants were indifferent between the two strategies. As in Gilbert et al. (2020), this is calculated by fitting a sigmoid curve to the strategy choices (0 = own memory; 1 = reminder) across the 8 target values (2–9) using the R package ‘quickpsy’ bounded to the range 2–9.
- Reminder bias: Difference between OIP and AIP (i.e. OIP – AIP). A positive value indicates a pro-reminder bias seeing as it shows that the participant used reminders at a lower value than OIP, i.e. a value where they would have earned more points with internal memory. A negative value indicates an anti-reminder bias, seeing as it shows that the participant did not use reminders when they would have earned more points by doing so.
- Prospective confidence: Initial pre-task confidence judgement
- Retrospective confidence: Final post-task confidence judgement
- Prospective metacognitive bias: Difference between prospective confidence and mean forced internal accuracy (positive = overconfident; negative = underconfident)
- Retrospective metacognitive bias: Difference between retrospective confidence and mean forced internal accuracy (positive = overconfident; negative = underconfident).

### Results

A summary of behavioural results is presented in Table 1. In line with previous experiments, accuracy in forced internal trials (no-feedback group: *M* = 65.06%, *SD* = 17.96; feedback group: *M* = 69.63%, *SD* = 16.38) was lower than forced external trials (no-feedback group: *M* = 97.01%, *SD* = 4.15; feedback group: *M* = 96.59%, *SD* = 5.71) in both groups. Moreover, despite the introduction of the computer overwriting mechanism in our experimental paradigm, participants were able to use their chosen strategy in well over half of the trials (no-feedback group: *M* = 73.40%, *SD* = 5.09; feedback group: *M* = 75.61%, *SD* = 5.69).

**Table 1:** Summary of behavioural results. Means of dependent variables are shown with standard deviations in parentheses

	No feedback	Feedback
Forced internal accuracy (ACC_FI_)	65.1 (18.0)	69.6 (16.4)
Forced external accuracy (ACC_FE_)	97.0 (4.2)	96.6 (5.7)
Pre-task confidence rating	47.2 (26.8)	58.6 (17.9)
Post-task confidence rating	58.6 (25.6)	63.6 (16.5)
Pre-task metacognitive bias	− 17.9 (27.6)	− 11.1 (17.6)
Post-task metacognitive bias	− 6.44 (19.5)	− 6.1 (12.4)
Pre-task absolute metacognitive bias	26.1 (19.8)	16.4 (12.8)
Post-task absolute metacognitive bias	16.2 (12.5)	11.4 (7.7)
Actual indifference point (AIP)	5.06 (2.57)	6.30 (2.47)
Optimal indifference point (OIP)	6.66 (1.71)	7.11 (1.46)
Reminder bias	1.60 (2.27)	0.81 (2.27)
Absolute reminder bias	2.30 (1.55)	1.91 (1.46)
Total reminders used	8.35 (2.71)	7.20 (2.62)

#### Influence of feedback on metacognitive bias

**Fig. 2:**
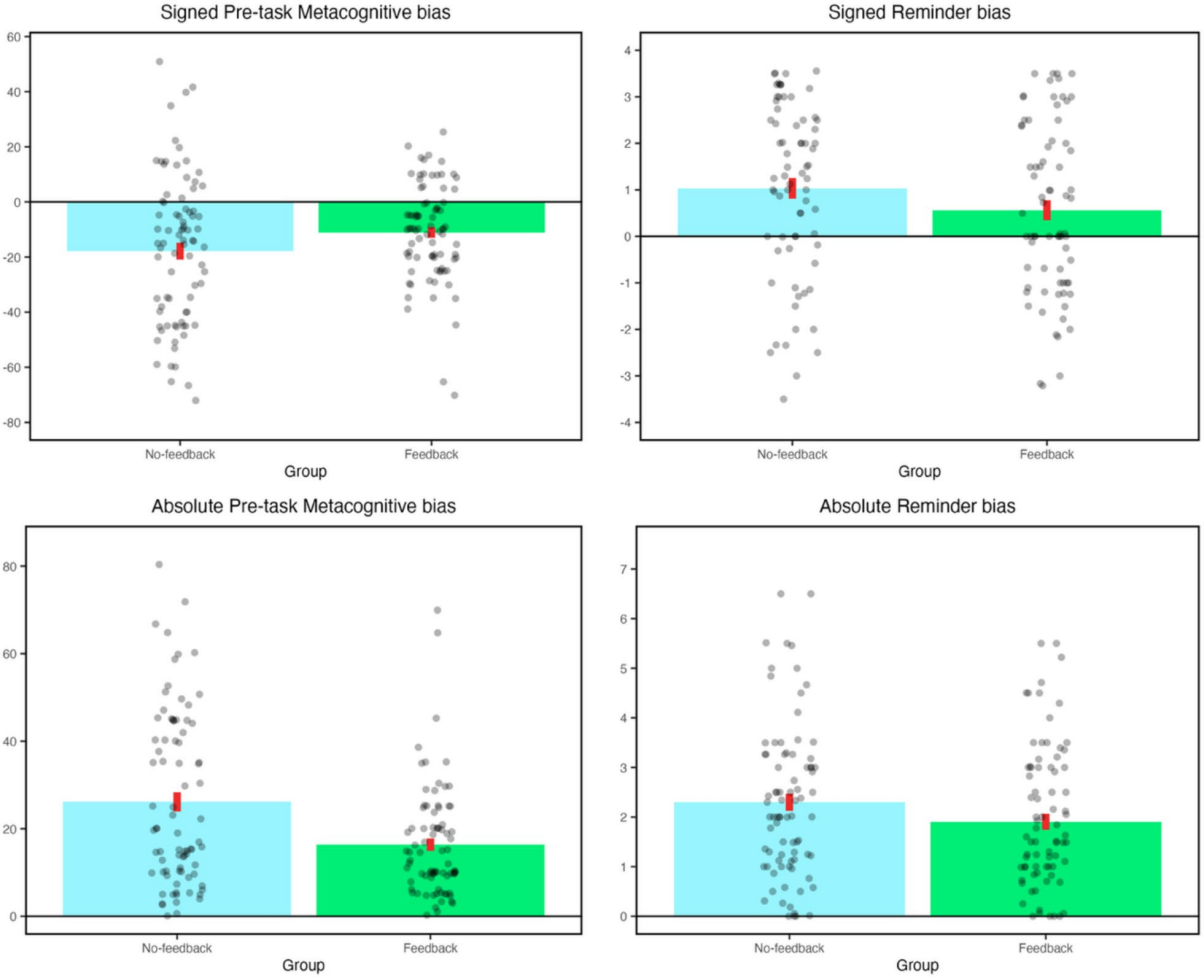
Key experimental findings. Grey dots indicate results from each individual participant. Red bars indicate standard error of the mean

Our first hypothesis concerned whether participants showed an internal metacognitive bias, and whether the provision of feedback improved the accuracy of metacognitive judgements. To investigate whether metacognitive bias scores differed according to feedback group and timepoint, we conducted a mixed 2 × 2 ANOVA with factors Group (feedback, no-feedback) and Time (pre-task, post-task). There was a significant main effect of Time (*F*(1,162) = 28.5, *p* < .001, η^2^_*p*_ = .15), with a reduction in underconfidence from pre-task (*M* = -14.5, *SD* = 23.3) to post-task (*M* = -6.3, *SD* = 16.3), showing that participants were less biased at the end of the experiment than the beginning (Fig. 2). Participants in both groups were significantly underconfident at both timepoints (*t*(81) > 2.9, *p* < .005, *d* > .33) and the reduction in underconfidence from pre- to post-task was significant in both groups (*t*(81) > 2.7, *p* < .01, *d* > .32). Therefore, underconfidence was significantly reduced but not eliminated for the post-task compared with the pre-task judgement.

We did not find a significant main effect of Group on metacognitive bias scores (*F*(1,162) = 1.7, *p* = .19, η^2^_*p*_ = .011), but there was a significant Time x Group interaction (*F*(1,162) = 4.3, *p* = .039, η^2^_*p*_ = .026). Participants in the no-feedback group showed a larger reduction in underconfidence from pre-task (*M* =  − 17.9, *SD* = 27.6) to post-task (*M* = -6.4, *SD* = 19.5) than participants in the feedback group (pre-task: *M* =  − 11.1, *SD* = 17.6; post-task: *M* = -6.1, *SD* = 12.4).

Next, we directly compared metacognitive bias scores between the feedback and no-feedback groups. As specified in our pre-registered plan, we did this separately for the pre- and post-task judgement in order to evaluate whether any immediate effect of the intervention was maintained at the end of the experiment. At pre-task, the feedback group was significantly less biased than the no-feedback group (*t*(137.4) = 1.9, *p* = .03, *d* = .29; note that this *p* value is for a one-tailed test, in accordance with our pre-registered hypothesis that feedback should reduce metacognitive bias). However, the groups did not differ significantly in their post-task metacognitive biases (*t*(137.1) = .144, *p* = .44, *d* = .022; one-tailed test as above). Therefore, the feedback intervention led to a significant reduction in underconfidence at the pre-task rating but not post-task.

We observed similar patterns in the equivalent analyses investigating raw metacognitive judgements (rather than metacognitive bias scores, which are relative to objective accuracy). First we examined confidence ratings in a mixed 2 × 2 ANOVA with factors Group (feedback, no-feedback) and Time (pre-task, post-task). Similar to results with the metacognitive bias scores, there was a significant effect of Time (*F*(1, 162) = 28.46, *p* < .01, η^2^_*p*_ = .149) and a significant Group x Time interaction (*F*(1, 162) = 4.33, *p* = .039, η^2^_*p*_ = .026). In this analysis, there was also a significant main effect of Group (*F*(1, 162) = 6.90, *p* = .01, η^2^_*p*_ = .041). As with the analysis of metacognitive bias, the groups differed significantly for pre-task confidence (*t*(141.24) = 3.19, *p* = .002, *d* = .497) but not post-task confidence (*t*(138.63) = 1.47, *p* = .144, *d* = .229); NB these *p* values are for two-tailed tests, because although the pre-registration indicated one-tailed tests for these analyses, we realised that the direction of predicted results was not clearly specified in advance. Finally, turning to within-group differences, two-tailed paired t-tests showed that the difference between pre- and post-task confidence was significant in both the feedback group (*t*(81) = 2.76, *p* = .007, *d* = .290) and no-feedback groups (*t*(81) = 4.59, *p* < .001, *d* = .435).

#### Influence of feedback on reminder bias

Two-tailed one-sample t-tests showed that participants in both the feedback group (*t*(81) = 3.22, p = .002, d = .367) and in the no-feedback group (*t*(81) = 6.35, p < .001, d = .702) were significantly biased towards reminders, showing that both groups chose to set reminders on more trials than was optimal. However, the magnitude of this reminder bias was significantly reduced in the feedback compared with the no-feedback group (*t*(162) = 2.22, *p* = .01, *d* = .347; note that this *p* value is for a one-tailed test, based on the pre-registered hypothesis that feedback should reduce the reminder bias). This result supported our hypothesis that metacognitive feedback would significantly reduce participants’ reminder bias, leading to more optimal reminder-setting behaviour (see Fig. 2).

#### Relationship between metacognitive bias and reminder bias

Our pre-registered analysis plan specified a Pearson correlation between the reminder bias and metacognitive bias, which suggested a negative correlation in the no-feedback group (*r*(80) = -.275, *p* = .006, one-tailed based on the pre-registered hypothesis that greater underconfidence should predict higher pro-reminder bias) but not the feedback group (*r*(80) = .068, no *p*-value calculated because a one-tailed test for a negative correlation was pre-registered). However, we subsequently realised that no conclusions can be drawn from these analyses, because both the reminder bias and the metacognitive bias were derived from the same underlying variable (forced internal accuracy), thereby violating the assumption of independence required for a Pearson test. Instead, we performed linear regression analyses predicting AIP (i.e. actual reminder strategy) from OIP (optimal reminder strategy) along with confidence rating (averaged over pre-task and post-task responses). In the no-feedback group, confidence explained significant unique variance (*t*(79) = 2.25, *p* = .027). Therefore, even after controlling for the optimal reminder usage strategy, which is calculated from actual levels of performance in the task, participants’ beliefs about their performance of the task were still significantly associated with offloading strategy, with lower confidence predicting higher offloading. However, in the feedback group, no such relationship between confidence and offloading strategy was observed (*t*(79) = .037, *p* = .97).

#### Additional, non-pre-registered analyses

Seeing as our intervention could potentially reduce both over- and underconfidence, we also performed additional analyses investigating the absolute values of metacognitive bias scores, i.e. the unsigned discrepancy between confidence and accuracy, regardless of whether confidence was lower or higher than accuracy. Absolute metacognitive bias was investigated in the same manner as the signed bias scores (see above). Consistent with the results for signed metacognitive bias scores, a mixed 2 × 2 ANOVA with factors Group (feedback, no-feedback) and Time (pre-task, post-task) revealed a significant main effect of Time (*F*(1,162) = 33.2, *p* < .001, η2ₚ = .17), with both groups showing a significant reduction in absolute metacognitive bias at post-task compared to pre-task (*t*(81) > 3.4, *p* < .01, *d* > .46).

However, in contrast with the findings with signed bias scores, we observed a significant main effect of Group (*F*(1, 162) = 17.6, *p* < .001, η2ₚ = .098) while the Group x Timepoint interaction was not significant (*F*(1, 162) = 3.68, *p* = .06, η2ₚ = .022). Direct comparisons between the feedback and no-feedback groups showed a significant group difference in both pre-task (*t*(138.1) = 3.76, *p* < .01, *d* = 0.59) and post-task (*t*(134.67) = 2.99, *p* < .01, *d* = 0.47) absolute metacognitive bias. At both time points, the feedback group (pre-task: *M* = 16.4, *SD* = 12.7; post-task: *M* = 11.4, *SD* = 7.7) was significantly less biased than the no-feedback group (pre-task: *M* = 26.1, *SD* = 19.8; post-task: *M* = 16.2, *SD* = 12.5).

Hence, while a significant group difference emerged only for pre-task metacognitive bias when using signed scores, the groups differed at both timepoints when comparing absolute metacognitive bias scores. Collectively, these results demonstrated that our metacognitive intervention was effective in reducing metacognitive bias.

Next, we conducted analogous analyses using the absolute values of reminder bias scores. We performed a one-tailed test, in alignment with the pre-registered analysis of the signed reminder bias score. Results were consistent with those observed for signed reminder bias scores: there was a significant reduction in bias in the feedback relative to the no-feedback group (*t*(161.40) = -1.67, *p* = .048, *d* = 0.26). This finding supported our prediction that metacognitive feedback promotes more optimal offloading behaviour.

### Discussion

This pre-registered study investigated the effect of a brief metacognitive training intervention on cognitive offloading strategies, using an ‘optimal offloading’ paradigm that permits an objective measure of each participant’s decision-making bias relative to their optimal strategy. In contrast with previous studies that have produced equivocal results (Engeler & Gilbert, 2020; Grinschgl et al., 2020), we found clear evidence that the training intervention reduced bias in both metacognitive judgements and offloading decisions, seeing as both the metacognitive bias and the reminder bias were significantly reduced in the feedback compared with the no-feedback group. This suggests that interventions aimed at improving the calibration of metacognitive judgements can indeed promote more optimal cognitive strategies.

Although the results indicated the impact of a metacognitive intervention, the underlying mechanism for this impact is uncertain. Participants in the feedback group both made predictions and received feedback, comparing their predictions with their actual accuracy over 5 practice trials, whereas the no-feedback group completed the same practice trials without making predictions or receiving feedback. This raises the question of whether the act of making metacognitive predictions alone could reduce metacognitive bias—by increasing task engagement, for example—thereby influencing reminder bias in turn. Alternatively, it is possible that the reduced metacognitive and reminder biases in the feedback group could relate both to generating additional metacognitive predictions and to receiving feedback on them. To disentangle these effects, it would be necessary to include an additional control condition where participants generate metacognitive predictions but do not receive feedback (cf Rummel et al., 2013).

Moreover, it is also worth noting that the feedback manipulation in this study comprised of three distinct components: i) a reminder of the participant’s own prediction, ii) their actual performance (hereafter 'performance feedback'), and iii) whether they underestimated, overestimated or accurately estimated their memory ability (hereafter 'metacognitive feedback'). Exactly what constitutes feedback has been variously defined in the metacognitive training literature: some studies provide participants solely with performance feedback (e.g. accuracy scores, see Grinschgl et al., 2025 for an example), while others explicitly communicate the nature of a participant’s metacognitive bias, specifying whether they are over- or under-confident in their estimates, in addition to performance feedback (e.g. Engeler & Gilbert, 2020). It remains unclear whether the latter, more detailed and directional form of feedback is necessary for improving self-estimates, and if so, to what extent it improves one’s metacognitive judgements above and beyond receiving performance feedback alone. The purpose of Experiment 2 was to clarify the influences of these factors.

## Experiment 2

**Fig. 3:**
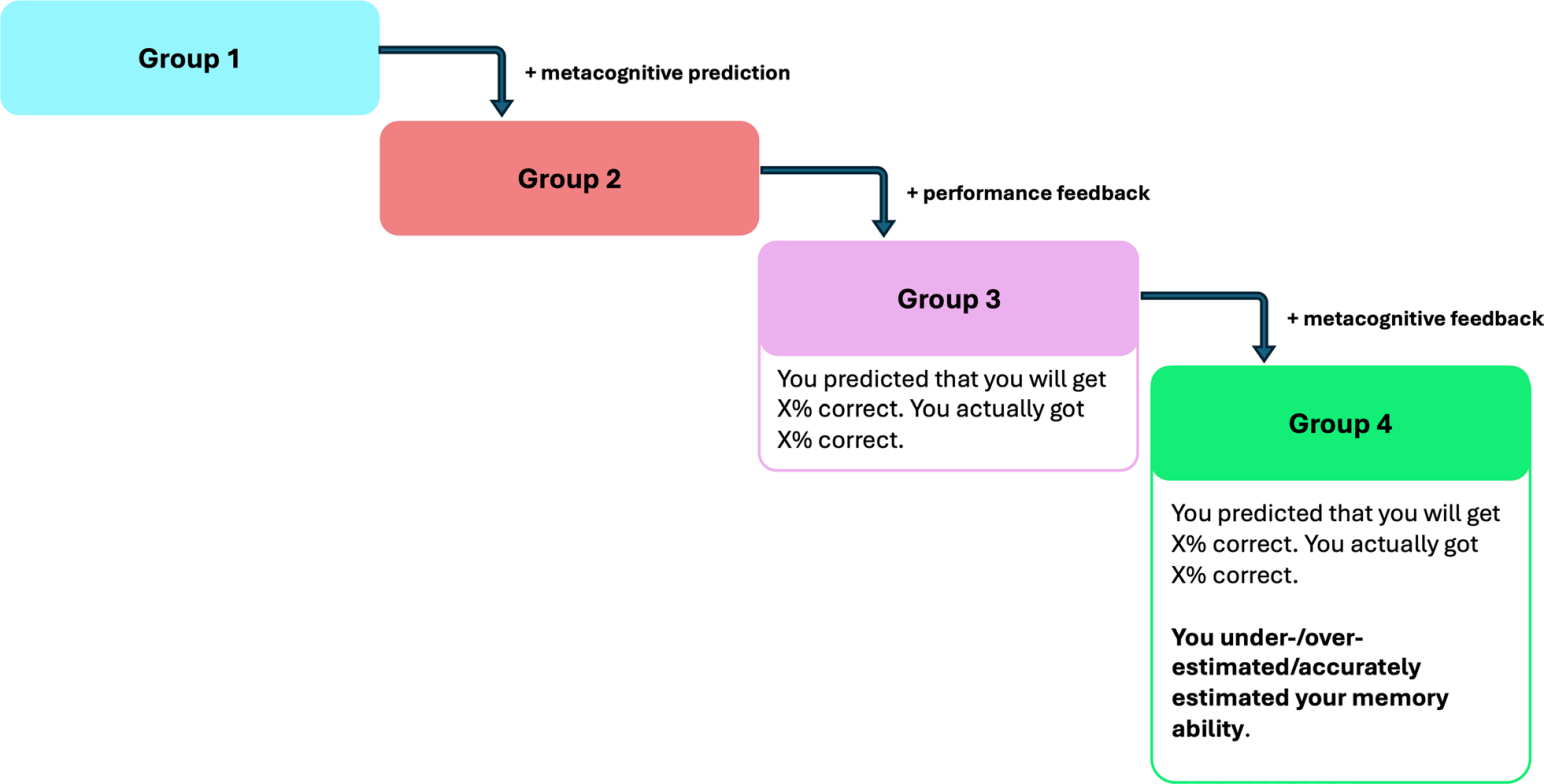
Illustration of experimental design for Experiment 2. Group 1 served as the control and was identical to the 'no-feedback' group in Experiment 1. Group 2 made metacognitive predictions without receiving feedback. Group 3 made metacognitive predictions and received performance feedback. Group 4 received the full intervention comprising both performance and metacognitive feedback, with the metacognitive feedback highlighted in bold text

This study aimed to provide further insight into how metacognitive training influences reminder setting. We replicated the experimental procedures of Experiment 1 with two additional groups of participants: a 'prediction, no-feedback' group and a 'prediction, performance feedback' group. Together with the replication groups, this resulted in a four-group design. A key feature of Experiment 2 was that the group conditions were designed to be *additive* (see Fig. 3). This meant that each successive group received all the interventions given to the previous groups, along with an additional component. Specifically, we began with Group 1 as the control and systematically introduced three factors across groups—whether participants made metacognitive predictions, whether they received feedback, and the specific type of feedback provided—with Group 4 receiving the full intervention incorporating all components (i.e. not only feedback about the original prediction and the actual accuracy, but whether this indicated over- or underconfidence). This additive design enabled us to isolate the effect of each intervention component, which in turn allowed us to address two outstanding questions from Experiment 1.

Hence, our main questions for Experiment 2 are:

- Can the key findings from Experiment 1 be replicated? Specifically, does the full metacognitive intervention reduce metacognitive and reminder biases?
- How do individual components of our experimental manipulation—making metacognitive prediction, receiving performance feedback, and receiving metacognitive feedback—influence metacognitive and reminder biases, respectively?

We predicted that the linear effect, testing for a progressive decrease in bias scores across the four groups, would be significant for both bias scores. All hypotheses, experimental procedures, and analysis plans were pre-registered prior to data collection (https://osf.io/d6b37/).

### Methods

#### Participants

We powered this study to replicate our earlier finding of a reduced reminder bias in the feedback versus no-feedback group (*t*(162) = 2.22, *p* = .01, *d* = .347). A sample of 208 participants (104 per group) was required to achieve 80% power to replicate this effect in a one-tailed, independent samples t-test (alpha = .05, *d* = .347, G*Power 3.1). This yielded a total sample of 416 participants for the current four-group design.

We tested a total of 506 participants to reach the planned sample size after applying our pre-registered exclusion criteria. These included: (a) forced internal accuracy below 10% (n = 2), (b) forced external accuracy below 70% (n = 30), (c) negative correlation between target value and likelihood of choosing to use reminders, suggesting random or counter-rational strategy choice (n = 36), (d) higher accuracy on forced internal than forced external trials (n = 4), (e) reminder bias score more than 2.5 Median Absolute Deviation units from other participants within their group (n = 6), (f) metacognitive bias score more than 2.5 Median Absolute Deviation units from other participants within their group (n = 12).

Our final sample consisted of participants aged 19 to 75 years (*M* = 35.2, *SD* = 11.4) including 241 men and 175 women. All participants were recruited via Prolific and took part by accessing a weblink on their own devices. The experiment was advertised with a base payment of £2.75 and an additional £1 bonus for performance in the top 50% on the offloading task. Participants provided informed consent and the study was approved by the UCL Research Ethics Committee (1584/003).

#### Procedure

The experimental procedures were mostly identical to Experiment 1. In addition to using an additive, four-group design, we also implemented the following modifications:

First, participants in the full intervention group (Group 4) now received feedback with the metacognitive element highlighted in bold text. This change was based on recent findings from Saenz et al. (2019), who demonstrated that salient feedback improves the accuracy of self-estimates in laboratory settings. During the metacognitive training phase, participants in Group 4 were presented with the following screen after each practice trial:

“You predicted that you will get X% correct. You actually got X% correct. **You underestimated/overestimated/accurately estimated your memory ability**.”

Furthermore, as an exploratory question, we also investigated how accurately participants remembered their initial, prospective metacognitive judgement, and whether they exhibited any memory distortions, such as a metacognitive hindsight bias. After completing the experimental trials, but before providing their post-task metacognitive judgement, participants were asked to recall their initial confidence report. The instructions were presented as follows:

“Remember that we asked you this question at the beginning of the experiment:

You have now completed this set of practice trials.

Before continuing, we would like you to tell us how **confident** you are that you can accurately perform the task for the rest of the experiment.

Please use the scale below to indicate what percentage of the special circles you can correctly drag to the instructed side of the square, on average. 100% would mean that you always get every single one correct. 0% would mean that you can never get any of them correct.

We would now like you to recall the answer that you originally gave to this question. Please tell us what answer you gave to this question when we asked you at the beginning of the experiment.

Please tell us what you said originally **NOT** how well you think you actually did in the experiment.”

#### Data analysis

All analyses were conducted using R (version 4.4.1). Data and analysis code can be accessed at https://osf.io/d6b37/. It should be noted that we failed to pre-register the analyses on the unsigned (i.e. absolute) metacognitive and reminder bias scores; however, we report these results alongside the signed bias scores in the same manner as our analyses from Experiment 1. Other key dependent measures were the same as Experiment 1, with one additional measure—metacognitive hindsight bias.

In our pre-registration, we operationalised metacognitive hindsight bias as the difference between remembered confidence and pre-task metacognitive judgement. Our analysis plan specified a 2 × 4 mixed ANOVA with within-subject factor Time (pre-task metacognitive judgement, recalled metacognitive judgement) and between-subject factor Group. However, we realised that this approach only tested whether participants remembered being more or less confident than they actually were at pre-task, rather than testing whether recalled confidence was shifted in the direction of actual performance (whichever direction that might be). A key feature of hindsight bias is that it involves memory distortions which occur *after the outcome is known* (Ackerman et al., 2020; Zimdahl & Undorf, 2021). Therefore, we revised our measure of hindsight bias to better reflect the relationship between confidence and performance:

- If actual accuracy was greater than initial confidence, then hindsight bias was calculated as subsequently remembered confidence minus initial confidence;
- If actual accuracy was lower than initial confidence, then hindsight bias was calculated as initial confidence minus subsequently remembered confidence

This updated measure now captures directional memory distortions, where a positive value indicates misremembering in the direction of actual performance.

### Results

See Table 2 for a summary of behavioural results. Performance on the task was consistent with Experiment 1, with participants achieving higher accuracy in forced external trials (Group 1: *M* = 96.35%, *SD* = 5.71; Group 2: *M* = 97.12%, *SD* = 5.15; Group 3: *M* = 96.68%, *SD* = 5.42; Group 4: *M* = 97.07%, *SD* = 5.29) than in forced internal trials (Group 1: *M* = 63.99%, *SD* = 19.76; Group 2: *M* = 68.61%, *SD* = 19.14; Group 3: *M* = 67.93%, *SD* = 17.02; Group 4: *M* = 66.83%, *SD* = 18.85). Moreover, participants were again able to apply their chosen strategy in over half of the trials (group 1: *M* = 74.8%, *SD* = 4.88; group 2: *M* = 74.3%, *SD* = 4.79; group 3: *M* = 75.7%, *SD* = 5.5; group 4: *M* = 74.7%, *SD* = 5.74).

**Table 2:** Summary of behavioural results

	Group 1	Group 2	Group 3	Group 4
Forced internal accuracy	63.99 (19.76)	68.61 (19.14)	67.93 (17.02)	66.83 (18.85)
Forced external accuracy	96.35 (5.71)	97.12 (5.15)	96.68 (5.42)	97.07 (5.29)
Actual indifference point	5.76 (2.90)	6.24 (2.71)	6.55 (2.59)	5.63 (2.60)
Optimal indifference point	6.56 (1.84)	6.94 (1.75)	6.93 (1.53)	6.76 (1.67)
Pre-task confidence	58.49 (26.31)	55.5 (28.93)	58.43 (21.78)	55.19 (18.36)
Post-task confidence	61.99 (24.22)	65.03 (24.08)	59.36 (20.69)	56.39 (20.94)
Recalled confidence	64.5 (24.2)	63.7 (27.9)	62.8 (21.8)	62.3 (18.6)
Pre-task metacognitive bias	− 5.5 (30.9)	− 13.1 (32.1)	− 9.5 (21.2)	− 11.6 (21.1)
Post-task metacognitive bias	− 2 (27.0)	− 3.58 (22.9)	− 8.58 (19.1)	− 10.4 (17.4)
Pre-task (Absolute) metacognitive bias	25.4 (18.27)	28.22 (20.01)	17.54 (15.14)	19.37 (14.25)
Post-task (Absolute) metacognitive bias	22.13 (15.51)	18.75 (13.47)	16.42 (12.88)	16.66 (11.46)
Total reminders used	7.83 (2.93)	7.31 (2.75)	6.97 (2.63)	7.81 (2.63)
Reminder bias	0.79 (2.83)	0.71 (2.82)	0.38 (2.41)	1.13 (2.28)
Absolute reminder bias	2.42 (1.65)	2.29 (1.79)	1.86 (1.58)	1.99 (1.58)
Hindsight bias	4.68 (15.56)	9.18 (20.37)	6.63 (20.39)	5.72 (15.19)

Means of dependent variables are shown with *standard deviations in parentheses*

#### Effect of intervention on prospective (pre-task) metacognitive bias

We first investigated whether the metacognitive intervention reduced pre-task metacognitive bias (i.e. difference between prospective metacognitive judgement and true forced internal accuracy) across the four groups. We examined both absolute metacognitive bias (deviation from perfect calibration) and signed metacognitive bias (degree of over- or underconfidence). Our key analysis consisted of a one-way ANOVA with linear contrast across the four groups, followed by a one-tailed t-test comparing Group 1 with Group 4 to replicate the findings from Experiment 1. We predicted a significant effect of the linear contrast.

Analysis of signed metacognitive bias scores did not show a significant group effect (*F*(3, 412) = 1.58, *p* = .194), nor a significant linear trend (*F*(1, 412) = 1.58, *p* = .209). Numerically, participants in Group 4 (*M* = -11.6, *SD* = 21.1) actually exhibited greater underconfidence than those in Group 1 (*M* = -5.5, *SD* = 30.9). Thus, the results for signed metacognitive bias scores did not support our directional hypothesis.

We conducted equivalent analyses using absolute metacognitive bias scores. The ANOVA revealed a significant effect of group (*F*(3, 412) = 8.98, *p* < .001, ${\eta }_{p}^{2}$ = 0.06). In line with our prediction, the linear contrast was significant (*F*(1, 412) = 14.79, *p* < .001, ${\eta }_{p}^{2}$ = 0.03). Moreover, the planned comparison between Group 1 and Group 4 was also significant (*t*(194.49) = 2.66, *p* = .004, *d* = 0.37). That is, participants who were given the full intervention (corresponding to the feedback group in Experiment 1) were significantly less biased than the control group (corresponding to the no-feedback group in Experiment 1). Therefore, although the signed bias findings from Experiment 1 were not replicated, the unsigned bias findings were.

To further characterise the reduction in absolute metacognitive bias scores, we performed one-tailed t-tests for all pairwise group comparisons, testing for a progressive decrease in bias from Group 1 to Group 4. In addition to the previously reported Group 1 versus Group 4 comparison, we observed significant reductions in absolute metacognitive bias for the following group pairs: Group 1 versus Group 3 (*t*(199.13) = 3.38, *p* < .001, *d* = 0.47), Group 2 versus Group 3 (*t*(191.79) = 4.34, *p* < .001, *d* = 0.60), and Group 2 versus Group 4 (*t*(186.09) = 3.68, *p* < .001, *d* = 0.51). There were no significant group differences between Group 1 and Group 2, or between Group 3 and Group 4. Therefore, these findings suggest that the key transition occurred between Group 2 and Group 3.

Taken together, these findings present three key implications. First, metacognitive training is effective in reducing absolute metacognitive bias, as evidenced by the significant linear contrast and pairwise comparisons. Second, making metacognitive predictions alone did not significantly improve metacognitive calibration (Group 1 versus Group 2). Third, although performance feedback was sufficient to improve metacognitive calibration (Group 3 versus Group 2), metacognitive feedback (i.e. explicitly highlighting over- or underconfidence) did not lead to any significant additional effect (Group 4 versus Group 3).

#### Effect of intervention on retrospective (post-task) metacognitive bias

Given the significant results on prospective metacognitive bias, we next conducted analogous analyses using retrospective (post-task) metacognitive bias, to assess whether the effects observed at pre-task persisted until the end of the experiment. Since neither of our tests for signed prospective metacognitive bias were significant, we focused exclusively on absolute bias in these analyses, in accordance with our pre-registered approach. Consistent with the prospective results, the ANOVA revealed a significant overall effect of Group (*F*(3, 412) = 4.04, *p* = .007, ${\eta }_{p}^{2}$ = 0.03) as well as a significant linear effect (*F*(1, 412) = 10.16, *p* = .002, ${\eta }_{p}^{2}$ = 0.02). In addition, a one-tailed, independent samples t-test also confirmed that participants in Group 4 were less biased than those in Group 1 (*t*(189.67) = 2.89, *p* = .002, *d* = 0.4). Therefore, the effect of our metacognitive intervention was evident at both timepoints; that is, improvements in metacognitive calibration were maintained at the end of the experiment.

Pairwise comparisons showed a largely similar pattern to the prospective bias results. However, there was now a significant difference between Group 1 and Group 2 (*t*(202.04) = 1.68, *p* = .047, *d* = 0.23), whereas the Group 2 versus Group 3 and Group 2 versus Group 4 comparisons were no longer significant. All other comparisons replicated the prospective bias findings.

#### Effect of intervention on reminder bias

Turning to reminder bias (i.e. difference between Optimal Indifference Point and Actual Indifference Point; see ‘Data Analysis’ in Experiment 1), we performed parallel analyses to examine whether the metacognitive intervention influenced participants’ use of reminders. We predicted that participants would set reminders more optimally (i.e. show reduced reminder bias) with the introduction of additional intervention components. As with metacognitive bias, we investigated both signed reminder bias (under- or over-reliance on reminders) and absolute reminder bias (distance from the most optimal strategy choice).

**Fig. 4:**
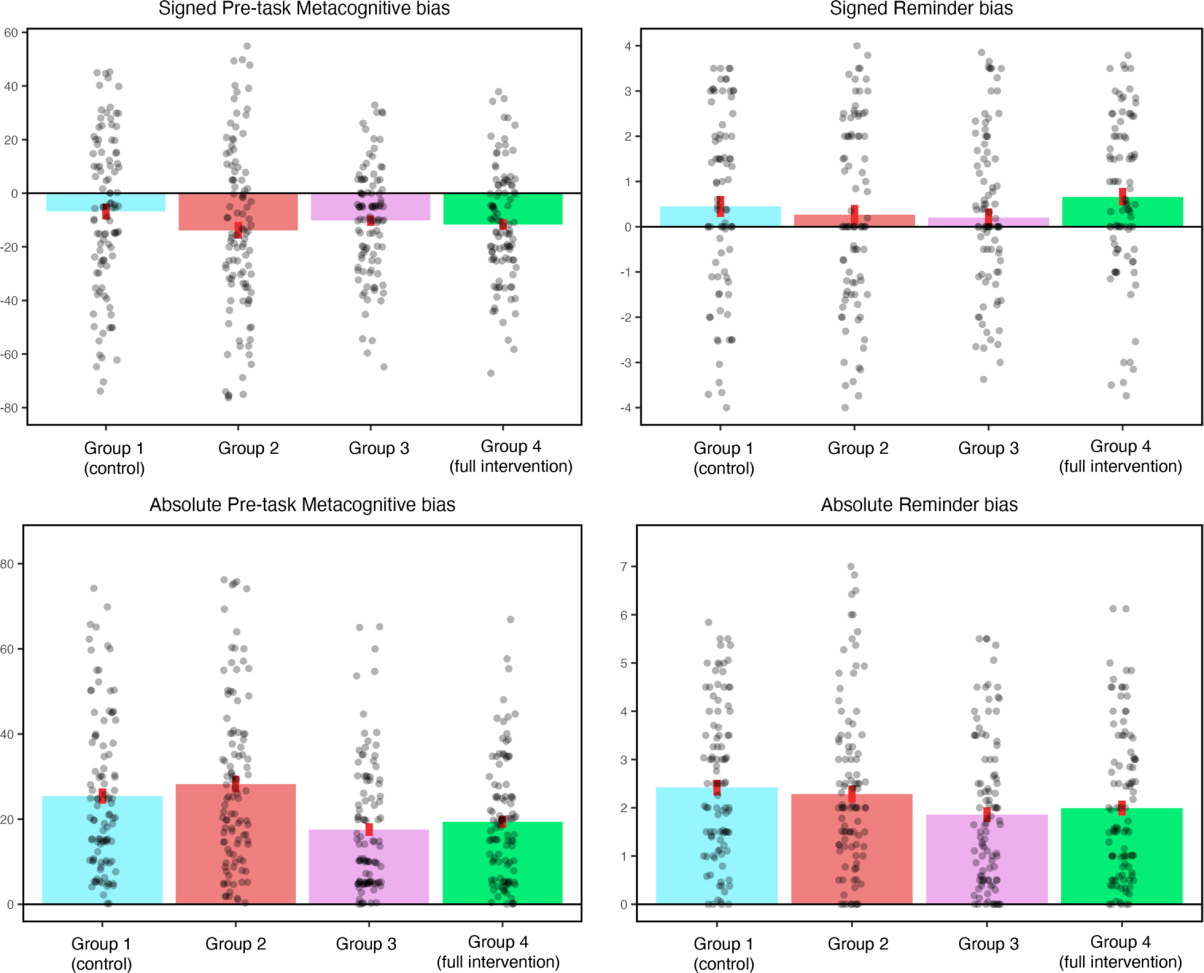
Key experimental findings. Grey dots indicate results from each individual participant. Red bars indicate standard error of the mean

Similar to findings from signed metacognitive bias, analysis of signed reminder bias did not yield significant effects. The ANOVA did not produce a significant effect of Group (*F*(3, 412) = 1.46, *p* = .224), nor was the linear contrast significant (*F*(1, 412) = 0.37, *p* = .546). The direct comparison between Group 1 and Group 4 produced an effect in the opposite direction to the pre-registered one-tailed test. Thus, with respect to signed reminder bias, our metacognitive intervention did not lead to the predicted improvements in reminder-setting behaviour (Fig. 4).

Next, we conducted equivalent analyses using absolute reminder bias. Although the ANOVA did not show a significant overall effect of Group (*F*(3, 412) = 2.56, *p* = .055), the linear contrast was significant (*F*(1, 412) = 5.58, *p* = .02, ${\eta }_{p}^{2}$ = 0.01), showing that one or more elements of our metacognitive intervention led to more optimal offloading behaviour. Moreover, the planned comparison between Group 1 and Group 4 replicated results from Experiment 1 (*t*(205.62) = 1.91, *p* < .05, *d* = 0.26): participants who received the full intervention (Group 4) showed significantly improved reminder strategy selection compared to the control group (Group 1). These results support our hypothesis that metacognitive training significantly reduces bias in reminder setting.

**Fig. 5:**
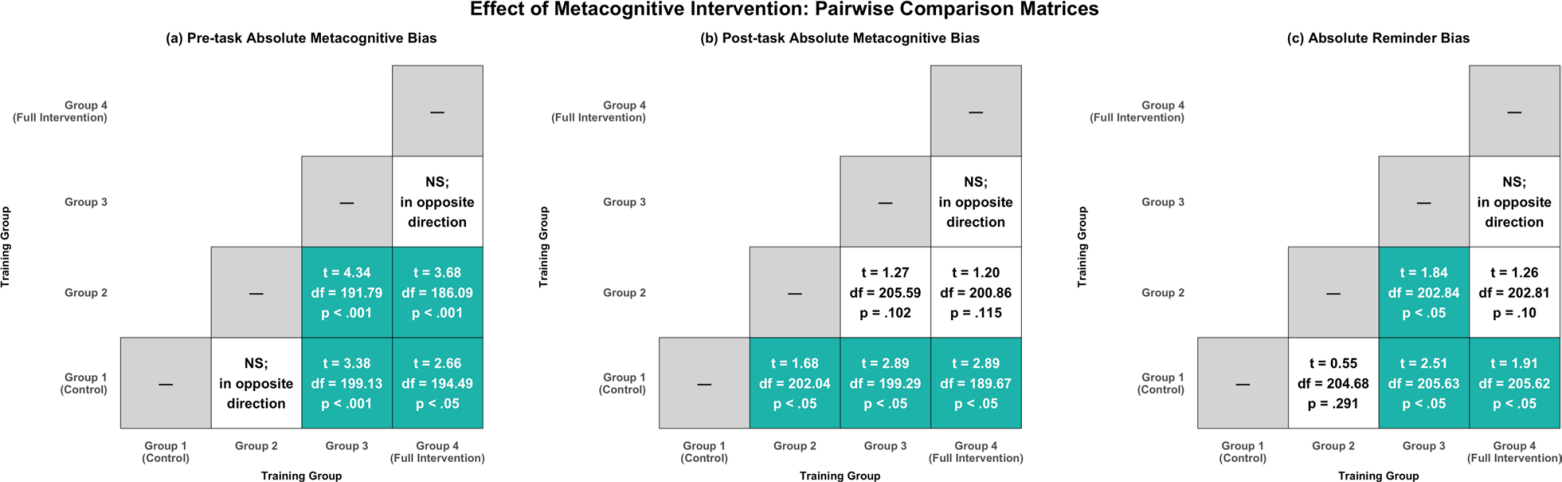
Overview of pairwise comparisons for (a) pre-task metacognitive bias, (b) post-task metacognitive bias, and (c) reminder bias. Note that all comparisons were one-tailed. Cells labelled ‘NS; in opposite direction’ reflect results that were contrary to our pre-registered hypothesis, so one-tailed significance testing was not appropriate

Pairwise comparisons revealed a pattern similar to that observed for absolute metacognitive bias. Specifically, insofar as adjacent group pairs were concerned, only the comparison between Group 2 and Group 3 showed a significant group difference (*t*(202.84) = 1.84, *p* = .034, *d* = 0.25). Receiving metacognitive feedback (Group 4) in addition to performance feedback (Group 3) again led to no further improvement in reminder bias. Consistent with the results for absolute metacognitive bias, the Group 1 versus Group 3 comparison was also significant (*t*(205.63) = 2.51, *p* = .006, *d* = 0.35). Figure 5 presents an overview of pairwise comparisons for absolute prospective metacognitive bias, retrospective metacognitive bias, and reminder bias.

Collectively these findings provide strong evidence that metacognitive training can improve metacognitive calibration and facilitate optimal reminder use. Importantly, the greatest benefit was observed between Groups 2 and 3 for both metacognitive and reminder biases, suggesting that interventions which instruct participants to make metacognitive predictions and provide performance feedback may be the most effective.

#### Metacognitive hindsight bias

A two-tailed, one-sample t-test revealed a significant hindsight bias, *t*(398) = 7.26, p < .001, *d* = .36, suggesting that participants’ memory of their pre-task confidence was distorted in the direction of their actual performance. To investigate whether metacognitive hindsight bias differed across the four groups, we conducted a one-way ANOVA with Group as the between-subject factor, which yielded no significant main effect of Group (*F*(3, 395) = 1.15, *p* = .328). We then performed one-sample *t*-tests comparing metacognitive hindsight bias against zero separately for each group. This confirmed that participants in all four groups displayed a significant metacognitive hindsight bias (Group 1: *t*(103) = 3.01, *p* = .003, *d* = 0.30; Group 2: *t*(103) = 4.45, *p* < .001, *d* = 0.44; Group 3: *t*(103) = 3.22, *p* = .002, *d* = 0.32; Group 4: *t*(103) = 3.41, *p* < .001, *d* = 0.33). Hence, while participants showed a robust hindsight bias regarding their pre-task confidence, this bias was not significantly affected by metacognitive intervention in this experiment.

#### Relationship between metacognition and offloading strategy

In a final set of analyses, which were not pre-registered, we investigated the relationship between individual differences in confidence and offloading strategy measures. As in Experiment 1, we performed linear regression analyses predicting AIP (i.e. actual reminder strategy) from OIP (optimal reminder strategy) along with confidence rating (averaged over pre-task and post-task responses). In all four groups, the confidence measure explained significant unique variance (*t*(79) > 2.7, *p* < .008), in each case with lower confidence predicting greater reminder usage. Therefore, beliefs about performance explained significant variance in offloading strategies even after controlling for the optimal strategy implied by objective performance levels.

### Discussion

Experiment 2 found strong evidence that the brief metacognitive training intervention developed in Experiment 1 improved both metacognitive and reminder biases. The additive design offered further insight into the contributions of each element. While making predictions alone did not yield significant improvements in prospective metacognitive or reminder biases, both biases were reduced when participants additionally received feedback on their actual accuracy. Although there was a significant difference between Group 1 and Group 2 in the post-task metacognitive bias, this is hard to interpret in the absence of a significant pre-task effect, so requires replication before any conclusions can be drawn. Further metacognitive feedback in the form of an explicit statement whether participants had been over- or underconfident did not confer any significant additional benefit. Therefore, our results demonstrate that metacognitive training which combines metacognitive predictions with performance feedback may be the most effective and efficient.

It is worth noting that the significant effects for both metacognitive and reminder biases were observed only for absolute bias scores, but not for the signed bias scores that we pre-registered as our main outcomes of interest. Therefore, the metacognitive intervention in this study did not reliably lead to increased or decreased confidence, nor did it reliably lead to increased or decreased bias towards reminders. Instead, the effect was seen on a participant-specific level, whereby confidence tended to increase in underconfident individuals and decrease in overconfident individuals. The absence of effects in the signed bias scores might simply reflect a lack of power to detect such effects, seeing as an intervention that corrected equivalent under- and overconfidence in a pair of participants would perfectly cancel out in the signed bias scores but still be observable in the absolute biases. Another possibility is that the control group in Experiment 2 was more balanced between over- and under-confident participants, and between pro- and anti-reminder biases, for example due to cohort effects. Indeed, the control group’s pre-task underconfidence was on average about three times as large in Experiment 1 than Experiment 2, and the pro-reminder bias was about twice as large. As shown in the scatterplots, these directional averages conceal large variation between individuals, with some participants in both experiments being highly under- or over-confident. This limits the ability to detect a directional influence of the intervention, but not necessarily an absolute effect.

Another difference between Experiment 2 and Experiment 1 is that we asked participants to recall their initial confidence judgement, which revealed a robust metacognitive hindsight bias. This finding corroborates previous research which demonstrated evidence of hindsight bias in Judgements of Learning (JOLs; see Zimdahl & Undorf, 2020). Interestingly, despite the observed reductions in metacognitive and reminder biases, in this experiment we did not find evidence that the metacognitive intervention influenced hindsight bias. Together, these results suggest that the present intervention was effective in improving metacognitive calibrations and reminder strategy selection, but it did not mitigate all forms of metacognitive bias. Future research could explore whether and how metacognitive hindsight bias may be reduced through separate targeted interventions.

## General Discussion

The use of external tools to support cognitive functioning, in particular memory, has become an integral aspect of everyday life. As the repertoire of cognitive tools continues to expand and become more accessible in the digital age, understanding how to foster more adaptive use of these tools carries broad practical implications. Across two experiments, we found converging evidence that a brief metacognitive intervention consisting of making metacognitive predictions and receiving feedback significantly improved participants’ confidence calibration and led to more optimal reminder setting. We found that an intervention combining performance predictions with feedback was effective, but making predictions alone did not lead to a significant reduction in bias.

Our findings corroborate existing evidence within the metacognitive training literature demonstrating that interventions targeting metacognitive calibration can indeed reduce metacognitive bias (Carpenter et al., 2019; Saenz et al., 2019). Critically, in contrast with prior works which found limited evidence for the influence of feedback interventions on downstream behaviour (Engeler & Gilbert, 2020; Grinschgl et al., 2020, 2025), our study offers clear evidence that the benefits of metacognitive training can translate to actual offloading strategy selection.

Several factors may account for this difference in findings. First, in the current study, participants’ financial compensation was determined by the optimality of their offloading strategies (cf. Engeler & Gilbert, 2020; Grinschgl et al., 2025; Miller & Geraci, 2011). This may have reduced variance in behaviour attributable to other factors, such as a preference to avoid cognitive effort (Sachdeva & Gilbert, 2020), suggesting that metacognitive interventions may be particularly effective in situations that minimise non-metacognitive influences on behaviour. Furthermore, we also ensured that when participants received feedback it was always veridical. This could have greater impact on offloading strategies than fake feedback (Grinschgl et al., 2020), which may be less likely to be believed.

The pattern of results from our study is particularly noteworthy in light of previous work (Grinschgl et al., 2025) which did not find improvements in metacognitive judgements despite presenting feedback in exactly the same manner. In Grinschgl et al.’s study, participants made performance predictions before completing two task blocks, with the feedback group receiving a single feedback statement in between (“Before conducting this task, you indicated to solve XX questions correctly. Actually, you solved XX questions correctly”). No significant improvements in metacognitive judgements were observed for three of the four cognitive domains investigated—calibration accuracy only improved for working memory. Instead, metacognitive judgements generally improved over time across both groups regardless of feedback, suggesting that practice effects, rather than feedback, contributed to increased calibration accuracy.

In contrast, in the present study participants completed five practice trials, each with immediate performance feedback linked explicitly to their prior prediction. This iterative training structure may have fostered more meaningful engagement and ongoing self-reflection. Indeed, as Saenz et al. (2019) argued, feedback interventions are most effective when feedback is salient and explicit, and when learners are encouraged to actively reflect on it. Future work should more systematically explore the potential causal pathways for these effects. Future work is also needed to systematically compare different training structures, for example, trial-by-trial versus block-wise feedback, and different numbers of prediction-feedback cycles, to determine optimal conditions for improving metacognitive judgements and promoting adaptive cognitive offloading. It would also be useful to systematically explore the impact of design choices (e.g. the number of targets presented on each trial) on the reliability of the experimental task itself. We used fewer targets per trial in the present study compared with previous work (5 targets, versus 10 in Engeler & Gilbert, 2020). This helped minimise the task duration but may have increased noise. Therefore, it would be helpful for future work to directly investigate the reliability of measures, such as these, that are derived from relatively low numbers of observations.

### Practical implications

Our findings highlight the potential of metacognitive interventions to improve real-life cognitive offloading strategies, which could in turn facilitate people’s ability to remember intentions, fulfil cognitive goals and act as effective cognitive agents. However, further work is clearly required to test this possibility, since there are several factors that could limit the transfer of the metacognitive effect demonstrated here to real-life settings.

The first factor to consider is that the memory task used in this study is clearly not fully representative of memory demands in everyday life. Compared with the intentions studied in our experimental task, real-world intentions are typically held over a longer time period, have personal significance to the individual, and may have multiple opportunities for fulfilment rather than just a single moment. As a result, it is not clear that the present findings would generalise to offloading strategies for naturalistic intentions. To investigate the similarity of intention offloading between laboratory and naturalistic settings, Scott and Gilbert (2024) studied participants’ offloading strategies for naturally occurring real-world intentions embedded in their everyday lives. This study found that the principles established in experimental studies of cognitive offloading did generalise to real life. Most significantly for the present work, Scott and Gilbert (2024) found that metacognition is linked to cognitive offloading of real-world intentions, as it is for intentions studied in the laboratory. Notably, participants were more likely to set reminders when they had low confidence, and this was linked to increased fulfilment of plans embedded within everyday life. Nevertheless, it remains to be seen whether metacognitive interventions delivered in a naturalistic setting would have similar effects to the one investigated here.

A second factor that could potentially differ between the experimental test presented here and a real-world metacognitive intervention is the time interval between delivering the intervention and the time at which it may have its impact. In the present study, the intervention was delivered immediately before the experimental trials. The impact of metacognitive interventions over longer time periods is unknown.

Another temporal factor to consider is the gap, within the metacognitive intervention itself, between making a performance prediction and receiving post-task feedback. Here, this was just a few seconds but in everyday life people often make predictions about their ability to fulfil intentions hours, days, or weeks in the future. There is therefore the possibility of a long interval between the time at which a confidence judgement is most relevant (the moment of deciding whether to set a reminder) and the time at which feedback is received (the eventual moment of intention fulfilment). This may be a particularly distinctive challenge for designing metacognitive training protocols for prospective memory compared with other sorts of task. Potentially, one way to bridge this temporal gap would be to remind individuals of their prior confidence at the time that performance feedback is received. Indeed, Experiment 2 showed evidence for a metacognitive hindsight bias, in which participants’ memory for their earlier confidence reports was systematically distorted over the course of the experiment. This highlights the relevance of finding interventions that can reacquaint individuals with their earlier beliefs at the time at which feedback is delivered.

A final factor relevant to developing real-world interventions is the extent of generalisation from one task to another. In the present study, the metacognitive intervention was performed via exactly the same task that was subsequently used for the experimental trials. It remains to be seen whether an intervention delivered in one domain or category of intention would propagate to offloading strategies in another domain. Previous work shows that cognitive offloading is influenced by both task-specific and domain-general metacognitive signals (Gilbert, 2015; Sachdeva & Gilbert, 2024), but the impact of a metacognitive intervention delivered in one domain on cognitive offloading in another has not yet been investigated. Some contemporary models of metacognition posit a hierarchy of metacognitive representations, from moment-by-moment confidence in individual decisions, up to task-level evaluations of performance and eventually global beliefs such as self-efficiency and self-esteem (Rouault et al., 2019; Seow et al., 2021). The bottom of this hierarchy aligns more closely with moment-by-moment metacognitive experiences such as affective or effort-related feelings, whereas the top corresponds with global beliefs. In the present study, the metacognitive interventions were delivered towards the bottom of this hierarchy, seeing as predictions and feedback related to a single trial of the experimental task (though note that the intervention could have been delivered at the even lower level of individual to-be-remembered items rather than a single trial composed of multiple items). Potentially, metacognitive interventions delivered at other levels of the metacognitive hierarchy could have distinct impacts, both in terms of their influence on the task at hand and also on the extent to which they generalise to distinct domains (cf Hoogervorst et al., 2025).

## Conclusion

In summary, this study showed that a brief metacognitive intervention can reduce bias in both metacognitive judgements about memory ability and also participants’ decisions about the optimal cognitive offloading strategy. Our findings hold promise for the development of effective metacognitive training programmes in applied settings and point to important avenues of future research. Promising directions include examining the influence of different training structures and investigating the generalisation of training effects across domains and to real-world settings.

## Data Availability

The original study pre-registration, data, and code to reproduce all analyses can be found at: https://osf.io/8yrew/(Experiment 1) and https://osf.io/g45aq (Experiment 2).
